# Costs and economic evaluations of Quality Improvement Collaboratives in healthcare: a systematic review

**DOI:** 10.1186/s12913-020-4981-5

**Published:** 2020-03-02

**Authors:** Lenore de la Perrelle, Gorjana Radisic, Monica Cations, Billingsley Kaambwa, Gaery Barbery, Kate Laver

**Affiliations:** 1 Department of Rehabilitation, Aged and Extended Care, Flinders University, Bedford Park SA, GPO Box 2100, Adelaide, 5001 South Australia; 2 Cognitive Decline Partnership Centre, the University of Sydney, Hornsby Ku-Ring-Gai Hospital, Hornsby, NSW Australia; 3 Health Economics, College of Medicine and Public Health, Flinders University, Bedford Park, SA Australia; 4 Health Services Management, School of Medicine, Griffith University, Southbank, Qld Australia

**Keywords:** Quality improvement, Collaborative, Cost, Cost-effectiveness, Healthcare, Guidelines, Implementation, Economic evaluation

## Abstract

**Background:**

In increasingly constrained healthcare budgets worldwide, efforts to improve quality and reduce costs are vital. Quality Improvement Collaboratives (QICs) are often used in healthcare settings to implement proven clinical interventions within local and national programs. The cost of this method of implementation, however, is cited as a barrier to use. This systematic review aims to identify and describe studies reporting on costs and cost-effectiveness of QICs when used to implement clinical guidelines in healthcare.

**Methods:**

Multiple databases (CINAHL, MEDLINE, PsycINFO, EMBASE, EconLit and ProQuest) were searched for economic evaluations or cost studies of QICs in healthcare. Studies were included if they reported on economic evaluations or costs of QICs. Two authors independently reviewed citations and full text papers. Key characteristics of eligible studies were extracted, and their quality assessed against the Consolidated Health Economic Evaluation Reporting Standards (CHEERS). Evers CHEC-List was used for full economic evaluations. Cost-effectiveness findings were interpreted through the Johanna Briggs Institute ‘three by three dominance matrix tool’ to guide conclusions. Currencies were converted to United States dollars for 2018 using OECD and World Bank databases.

**Results:**

Few studies reported on costs or economic evaluations of QICs despite their use in healthcare. Eight studies across multiple healthcare settings in acute and long-term care, community addiction treatment and chronic disease management were included. Five were considered good quality and favoured the establishment of QICs as cost-effective implementation methods. The cost savings to the healthcare setting identified in these studies outweighed the cost of the collaborative itself.

**Conclusions:**

Potential cost savings to the health care system in both acute and chronic conditions may be possible by applying QICs at scale. However, variations in effectiveness, costs and elements of the method within studies, indicated that caution is needed. Consistent identification of costs and description of the elements applied in QICs would better inform decisions for their use and may reduce perceived barriers. Lack of studies with negative findings may have been due to publication bias. Future research should include economic evaluations with societal perspectives of costs and savings and the cost-effectiveness of elements of QICs.

**Trial registration:**

PROSPERO registration number: CRD42018107417.

## Background

A significant challenge facing health care settings is how to implement proven clinical interventions in practice in a cost-effective manner [1]. Scarce resources, including lack of time and staff are often cited as barriers to implementation [2, 3]. A recent review of medical research shows health savings from broad research translation, significantly outweigh the cost of delivering them [3] but the field of economic evaluation of implementation strategies is still developing [4]. Decisions to use particular implementation methods can be better informed by identifying cost-benefits of methods in addition to health outcomes [5, 6].

Methods of knowledge translation have been tested with mixed results. For example, clinical practice guidelines aim to translate research into practice and improve the quality of care and health outcomes for people. However, studies have shown that the dissemination of guidelines alone is insufficient to effect change in routine clinical practice [7]. Education and training of clinicians, the development of champions of change in organisations, and audit and feedback mechanisms have been trialled to improve adherence to guidelines [8]. However, these strategies lead to only modest effects in quality improvement [8]. A recent review found that while multifaceted strategies are more effective, costs associated with components were difficult to discern and cost-effectiveness was not explicitly evaluated [9]. Knowledge translation approaches which are tailored to an organisation can be successful but may lack transferability to other settings [10–12]. QICs have been adapted from manufacturing industry [13] for use across multiple settings by the US Institute for Healthcare Improvement (IHI) [14]. A QIC is a multifaceted approach to implementation of evidence-based practices, clinical guidelines or improved methods for quality and safety. Typically, they draw participants from multiple healthcare organisations to learn, apply and share improvement methods over a year or more. Teams are supported by experts who coach participants to test strategies adapted to their own setting. By collaborating, participants learn more effectively, spread improvement ideas and benchmark their progress against other organisations [14, 15]. Common components of QICs include face to face training sessions focussing on healthcare improvement and quality improvement methods, telephone meetings, feedback and the use of process improvement methods [13]. QICs have been used in healthcare systems in several countries to improve implementation outcomes [15–17]. They are adaptable within complex healthcare systems and offer a way to scale up implementation across many different organisations. However, inconsistent results, multiple elements and perceived cost of establishing, conducting and sustaining a collaborative are barriers to their use [17–19]. Wells and colleagues recently identified 64 QICs reporting effectiveness measures that met their inclusion criteria [15]. They found that 73% of these collaboratives reported significant results in diverse settings such as hospitals, health clinics and nursing homes. Improvement was associated with targeted clinical practice related to infection control, management of chronic conditions or prevention of falls, wounds or pain management [15]. While these improvements were associated with cost savings, only four studies reported on cost-effectiveness outcomes [15]. They identified gaps in design, reporting and assessment of costs which limited the information on cost-effectiveness. The costs of establishing a QIC can be significant, including personnel to recruit and coordinate activities, development of materials and education, the time spent by all participants involved in the collaborative and expenses associated with face to face meetings [17].

With increasing pressure on the healthcare system to deliver evidence-based practice with scarce resources, there is a need to evaluate the cost-effectiveness of healthcare improvement and knowledge translation strategies. Economic evaluation can assess implementation strategies to guide decisions about the choice of strategy providing value for money.

The aim of this systematic review was to identify and describe studies that report on the costs and cost-effectiveness of QICs to inform strategies to implement clinical guideline recommendations in healthcare.

## Methods

The protocol for this systematic review was developed in advance and was registered with PROSPERO on 7 September 2018; registration number CRD42018107417.

### Eligibility criteria

Studies were included in this review if they reported on initiatives that comprised healthcare clinicians across teams, professions, or organisations involved in a QIC or a quality improvement team with the aim of improving practice over time. Quality improvement teams were included if they included the most common components of QICs as identified by Nadeem et al. [13]. Studies were included if the collaboratives used multi-modal interventions, such as training, developing implementation plans, trying out a practice improvement, seeking advice from experts and people with lived experience and reviewing plans over time to improve practice [15]. We included quantitative studies that used full economic evaluation (i.e. cost-effectiveness, cost-utility analysis, cost-benefit analysis, cost-consequences analysis); partial economic evaluations (i.e. cost analyses, cost descriptions, cost outcome descriptions, cost minimisation studies); and randomised trials reporting estimates of resource use or costs associated with implementation or improvement. We excluded systematic reviews, study protocols, conference proceedings, editorials and commentary papers, effectiveness analyses with no analysis of costs, burden of disease studies, and cost of illness studies. The primary outcome of interest was the cost-effectiveness or cost-benefit of the use of elements of QICs to implement improvement in healthcare or adherence to clinical guidelines. A secondary outcome was costs associated with QICs.

### Search strategy and study selection

Five electronic databases were searched on 19 November 2018 (CINAHL, Medline, PsycINFO, EconLit, ProQuest (Health and Medicine: Social Sciences subsets only)). Embase was searched on 20 August 2019. Websites of large organisations interested in healthcare improvement such as the Institute of Healthcare Improvement (IHI, USA) and government bodies such as National Health and Medical Research Council (Australia), National Health Services and the National Institute for Health and Care Excellence (UK) and the European Network of Health Economic Evaluation Databases were searched for grey literature. Reference lists of included studies were scanned for potentially eligible studies. Studies were limited to English language, but no time limits were imposed on the search strategies. Research librarians with expertise in systematic reviews assisted with the development of the search strategies. The search strategy was developed for MEDLINE using medical subject search headings (MeSH) and text words and then adapted for use with the other databases. The strategy combined terms relating to quality improvement, collaborative, guidelines implementation and cost, cost-benefit or economic analysis. The search strategy for MEDLINE is attached (Additional file 1). Results are reported per the Preferred Reporting Items of Systematic Reviews and Meta-Analyses (PRISMA) guidelines [20].

Two authors (LdlP and GR) independently screened titles and abstracts based on the inclusion criteria detailed in the review protocol. Full texts of studies identified by abstract and title screen as having met the inclusion criteria were obtained and reviewed independently (LdlP and GR). Differences between reviewer’s results were resolved by discussion and when necessary in consultation with a third review author (MC)**.**

### Data extraction

One author (LdlP) extracted data using a modified version of the Joanna Briggs Institute (JBI) Data Extraction form for Economic Evaluations [21]. Another author (GR) checked the extraction for accuracy. Data was extracted about the study method, evaluation design, participants, intervention used, comparator, outcomes, prices and currency used for costing, time period of analysis, setting, tools used to measure outcomes and authors conclusions. This information was presented descriptively and summarised in Table 1 (Additional file 2)*.* Both costs of care resulting from improved care and costs of establishing QICs were identified. Cost components were standardised by converting currency and year to US dollars for 2018 through the Eurostat-OECD data base and manual on purchasing power parities for Euros and The World Bank GDP deflator data base for United States dollar values [22, 23].

### Risk of bias assessment

Two checklists were used to critically appraise the studies due to the difference in design of studies included. The 24 item Health Economic Evaluation Reporting Standards (CHEERS) checklist was used to determine methodological quality of all the included studies as it applies to any form of economic evaluation [24]. This is presented in Table 2a (Additional file 3). The Evers CHEC-List [25] was also used to assess the full economic evaluations and is included as Table 2b (Additional file 4) [26]. A score of one point was assigned to each positive response, zero to a negative response or for items that did not apply. A summary score is calculated at the bottom of each table with a maximum score of 24 and 19 respectively. This scoring provides an indication of total items present for each study.

#### Assessment of generalizability

The currency and year of studies was converted to US dollars for 2018 using the Eurostat-OECD purchasing power parities data base for Euros and the World Bank deflator data base for US dollar updates. This provided an option to compare results but due to the varied type of studies and focus on the implementation method rather than the healthcare intervention, a full transferability assessment was not conducted.

### Data synthesis

Included studies were subjected to data extraction by the author (LdlP) and information was synthesised to interpret the findings of full and partial economic evaluations and cost analysis studies. The Johanna Briggs Institute (JBI) ‘three by three dominance ranking matrix tool’ was used to interpret findings [27] and was checked by another author (GR) for consistency. Any inconsistencies were resolved by discussion and by consultation with a third review author (BK). This tool assists in drawing conclusions about the results of studies in terms of both cost and effectiveness (health benefits). It classifies results as favoured, unclear or rejected in favour of the comparator. An intervention was favoured if relative to its comparator it either (i) was cheaper but more effective, (ii) was cheaper but just as effective or (iii) cost the same but was more effective. An intervention was rejected if, relative to its comparator, it either (i) was more expensive and less effective, (ii) was more expensive and just as effective or (iii) cost the same but was less effective. A judgement would have to be made about all other scenarios based on other criteria [27]. For instance, an intervention would be favoured if it was more expensive and more effective than a comparator provided the associated incremental cost-effectiveness ratio (ICER) was below the threshold used for assessing cost-effectiveness e.g. €80,000 per quality adjusted life years (QALY) in the Netherlands [28].

## Results

### Study selection

**Fig. 1:**
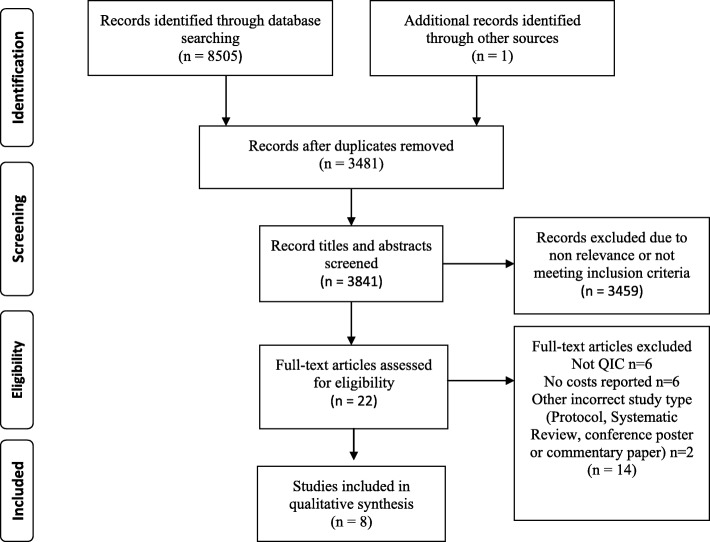
PRISMA flowchart describing the process of study selection

The search identified 8505 citations and after removing duplicates, 3481 titles and abstracts were reviewed. Twenty-two full text reviews revealed eight papers that met the inclusion criteria. PRISMA flowchart at Fig. 1 describes the process of selection [29].

### Overview of studies

Table 1 (Additional file 2) presents the overview of characteristics of the studies included in this review. Most studies describe the costs of establishing a collaborative to improve quality in healthcare and compared costs to outcomes. Five of the included studies involved full economic analyses using cost-effectiveness analysis (CEA) or cost utility analysis (CUA) [30–34], whereas three studies were cost analyses [35–37]. All studies were set in multi-centre healthcare settings, hospitals, long term care or community clinics, and related to diverse health conditions such as Parkinson’s disease, diabetes, obstetrics, neonatal intensive care, hip fractures, pressure ulcers, cardiac care or addiction treatment. All included clinicians working either nationally or across multiple states.

### Methodological quality

Table 2a (Additional file 3) summarises the methodological quality of the studies included in this review.

Cost effectiveness study conducted by Broughton et al. [30], and cost utility studies by Schouten et al. [33], Makai et al. [32] and Huang et al. [34] were considered high quality, complying with most of the items on CHEERS checklist [24]. Item 12 related to valuation of preferences for outcomes was not addressed in these studies [24]. A cost analysis by Bloem et al. [35] and a cost effectiveness study by Gustafson and colleagues [31] were of moderate quality. They did not address item 13, related to estimating costs via a model-based evaluation, items 15 and 16, the choice of model or assumptions or item 20, how uncertainty was addressed. The cost analysis by Rogowski et al. [36] was rated low quality on CHEERS checklist and the cost study by Dranove et al. [37] study was considered lowest quality as less than half of all items were addressed. Using the Evers CHEC-List [25], the full economic evaluations [30–34] were rated good quality.

Conflicts of interest and uncertainties in data were addressed by five studies [30, 32–35]. An incremental cost-effectiveness ratio (ICER) was not applicable for the cost analyses [35–37] and future costs were not directly considered for those studies.

### Data synthesis

Table 3 (Additional file 5) provides a three by three dominance ranking matrix (JBI DRM) tool to assist in interpreting the cost-effectiveness results of the studies included [27]. In this review, five studies were classified as favoured interventions (strong dominance) [30, 31, 33, 35, 36], two as unclear [32, 34] and one rejected [37]. Bloem et al. [35], Broughton et al. [30] and Schouten et al. [33] all showed reduced costs and improved health outcomes and are most favoured interventions. The studies by Gustafson et al. [31] and Rogowski et al. [36] show reduced costs for equally effective processes which are next favoured interventions. The study by Makai et al. [32] reported increased costs and reduced pressure ulcers while Huang et al. [34] reported that the improvements in Diabetes care were not cost-effective. These results are uncertain because while the interventions were more expensive but also cost effective, most scenarios analysed yielded ICERs that were above the traditionally accepted thresholds of €80,000/QALY [32] and US$100,000/QALY [34]. They therefore need to be assessed against specific priorities for health improvements and expenditure. In a cost analysis, Dranove et al. [37] were unable to identify cost savings or health improvements as a result of quality improvement expenditure and the comparator is favoured in this case.

### Effectiveness and cost-effectiveness

#### Clinical effectiveness

Five studies [30, 32–35] reported positive clinical outcomes as a result of using a QIC approach. In studies involving people with chronic health conditions, quality improvements led to reduced mortality risk and reduction in associated health events [33, 35]. For example, adherence to guidelines for Parkinson’s disease care achieved via the collaboratives produced improved outcomes, such as reduction in hip fractures, fewer hospital admissions, lower mortality risk and fewer disease related complications [35]. Quality improvement in diabetes care [33, 34] resulted in reduced scores for diabetes risk for cardiovascular disease events and mortality, reduced lifetime incidence of complications and improved life expectancy for both men and women. In both acute and critical care, the improvements led to reduced associated illness but differed in relation to the effect on mortality risk [30, 36]. In obstetric care, establishment of a QIC resulted in reduced post-partum haemorrhage, reduced mortality and increased numbers of births in clinics [30]. In neonatal intensive care, a QIC achieved reductions in infections in critically ill pre-term babies and reduced surgical interventions but no significant difference in mortality was found [36]. Residents in long term care had reduced incidence of pressure ulcers and slightly improved quality of life as a result of a QIC [32].

Gustafson and colleagues tested the effectiveness of four different elements of a QIC in the context of addiction treatment clinics [31]. This study compared clinic level coaching, group telephone calls to clinicians, face to face learning sessions and a combination of these elements to see which methods were more effective. This study did not collect patient outcomes but focussed on three primary process outcomes: waiting time, retention of patients and annual numbers of new patients. These process outcomes were chosen, as the link between treatment programs and patient outcomes was considered weak [31]. Significant improvements in waiting time and number of new patients were identified for two of the interventions: coaching and the combination of all three elements. A combination of all elements was found to be more costly than coaching alone although it was similarly effective [31]. Dranove and colleagues found no direct links between the clinical outcomes for patients of hospitals studied and the amount they spent on general quality improvement activities [37].

#### Cost-effectiveness and cost savings

Five studies [30, 31, 33, 35, 36] reported favourable cost findings from the use of QICs. These were related to savings in the health care system and did not consider broader costs and benefits such as lost productivity, non-medical patient costs and carer time. These studies are considered here in relation to cost effectiveness and cost savings achieved for the use of QICs across a range of health conditions and countries. Values provided below are conversions to US$ for 2018 [22, 23] where the price year was provided.

#### Cost-effectiveness

Within the context of diabetes care in the Netherlands [33], the QIC was found to be cost-effective. For the large populations of people who live with diabetes there are significant medical costs related to medicines and cardio-vascular disease [33, 34]. The incremental costs per quality adjusted life year (QALY) of US$1550–1714 compared favourably with other published studies on diabetes [33]. With a cost of about US$19 per patient for the QIC over 2 years, the cost-effectiveness was reported to be significant. In the US, a diabetes care improvement in public health clinics [34] found lower incidence of complications but the cost of individual improvements in care varied and all interventions but the use of an Angiotensin-converting enzyme (ACE) inhibitor, were not cost-effective [34].

The cost effectiveness study examining obstetric and newborn care in Niger [30] found the cost per normal delivery reduced, with a similar decrease in both numbers and costs of deliveries with post-partum haemorrhage [30]. The cost of the QIC was calculated to be US$2.84 per delivery. The incremental cost-effectiveness was US$335 per disability-adjusted life year (DALY) averted and the study concluded that if other obstetric clinics used the collaborative approach, substantive cost savings could be achieved [30].

In long term care [32], reduction in incidence of non-severe pressure ulcers using a QIC approach increased costs of care in the short term. Cost-effectiveness in the longer term was unclear due to small effects on quality of life in nursing home populations near the end of life, and the difficulty in sustaining trained staff to continue to prevent pressure ulcers. As a preventable condition however, quality improvement in the prevention and care of pressure ulcers for a vulnerable population was a worthy goal [32].

A comparison of four different approaches to implementing QICs (in the context of addiction treatment) identified cost-effective elements [31]. This study found that while both coaching and a combination of interventions were equally effective in reducing waiting times and increasing numbers of new patients there were significant differences in costs of the interventions. They found the estimated cost per clinic for a coaching intervention was US$2878 (no year) compared to US$7930 (no year) for the combination of interventions. They concluded that the coaching intervention was substantially more cost-effective [31].

#### Cost analyses

A cost analysis of ParkinsonNet [35] showed annual cost savings of US$449 per patient by avoiding or delaying complications or high cost treatments of Parkinson’s disease. The cost per patient per annum was around US$30. However, based on a population of 40,000 people with Parkinson’s disease in The Netherlands, they predicted a national cost saving of over US$17.4 million per annum as a result of the quality improvement [35].

In the costly area of neonatal intensive care, a cost analysis study [36] reported significant cost savings per infant were achieved. While costs varied, the average savings per hospital in the post intervention year was US$2.3 million for an average cost of $68,206 per hospital in resources to undertake the QIC [36].

Finally, the study of costs to improve quality of care in hospitals in United States [37], found a wide variety in expenditures on quality improvement activities which were not correlated with condition specific costs. Differences in costs were not statistically significant. They presumed that a lack of consensus about the purpose of quality improvement efforts at the time, led to this variation in costs and disconnection with outcomes [37].

#### Costs

##### Costs of care

The costs of clinical treatment were measured in most studies and included clinic visits or treatment provided in hospital such as ventilation, surgery and medications, complications or infections [30, 32–36]. Costs were extracted from hospital bills, medical claims and records maintained by clinicians. Some studies used estimations of costs to form their data, or surveyed managers to identify costs from budgets [30, 33]; one used weekly diaries of activities and applied hourly costs for personnel time [36]. Costs of care were not reported in two studies [31, 37].

##### Costs of establishing QICs

The most common costs identified were: program management costs for the QIC coordinators, time of the participating clinicians in face to face meetings, education sessions, collecting data, travel costs, conference calls, data analysis costs, overhead costs and some capital costs. The cost of developing evidence-based guidelines was included in the ParkinsonNet study to give a complete cost of start-up of the network [35]. Four studies provided a cost per patient of establishment of the QIC. These included US$3.67 per infant delivery [30], US$30 per person with Parkinson’s disease [35], US$19 per person with diabetes in Europe [33] and US$130 per patient with diabetes in USA [34]. Dranove et al. reported a wide variation in costs of quality improvement activities between hospitals with the highest costs attributed to meetings [37]. All reported costs are presented in Table 4 (Additional file 6).

## Discussion

There is a need for larger scale and more rapid translation of evidence-based interventions into practice [34]. However, the cost associated with research translation is an important consideration for constrained health care budgets. QICs have been used widely in diverse healthcare settings and have been effective in improving outcomes for patients [38] although the costs of the collaboratives may be a barrier to their use [35]. This review sought to identify and describe studies that report on the costs and cost-effectiveness of QICs in healthcare settings. Although a recent systematic review of QICs identified 64 studies on effectiveness, only four reported on cost-effectiveness [15]. We identified eight studies that reported on costs or cost-effectiveness of QICs. This included the four studies identified in the review by Wells et al. so updated that aspect of the review [15]. Our results confirm that the consideration of costs of QICs has not been reported in many studies. This may be because of the difficulty in defining costs associated with QICs over time and in different contexts [38, 39]. It may be that costs are small in comparison to operating costs or funded separately to the health system and of less importance for research [40].

Five of the eight studies in this review showed that QICs were cost-effective in implementing clinical guidelines [30, 31, 33, 35, 36]. They identified cost savings and improvement in health outcomes for patients in both acute care and chronic condition management. The costs associated with the QIC appeared low in relation to savings across large populations or for reducing the need for high cost treatments [36, 41]. These studies calculated the cost of the QIC per patient for the duration of the intervention which provided useful data compared to overall outcomes and savings achieved. Where smaller populations are treated with high cost interventions, the cost per patient for the QICs would be expected to be higher.

These studies were conducted in different countries or across states, with different infrastructure costs and resources. It would be difficult to generalize the costs of the QICs across such different countries and conditions. However, they used a similar process to engage clinicians and modify practices locally. This indicated that the QIC methodology was adapted to different conditions with similar set up structures needed. An investment in QICs was needed and the costs per person could be best spread across large populations of people with a condition or where high cost treatments can be reduced [38].

One study evaluated which element of the QIC intervention was more cost-effective [23]. This demonstrated that differences that can be achieved in both effectiveness and cost by the choice of how education or support was provided to clinicians. Only one study found no correlation between health outcomes and the costs of quality improvement activities in hospitals [26].

Although most of the studies captured only medical costs, most considered that societal effects of health improvements may increase the cost-effectiveness due to improved quality of life (QoL). For treatment of chronic conditions, improved care is likely to result in long term cost savings, however QoL in long term care populations was more difficult to measure [32]. Schouten et al. [22] found that a wide range of disease risk control was achieved in diabetes treatment. They suggested that outcomes of other chronic conditions may be improved through a QIC approach and the societal effects may also be higher when considering better quality of life outcomes. Bloem et al. [23] similarly identified the potential for improvement of cost-effectiveness of healthcare for other chronic disorders. They also reported the need to structure funding sources and medical insurance related to improvements in health outcomes.

Rogowski et al. [24] identified the potential for higher cost savings for expensive health interventions and at least short-term sustainability of QICs. Widespread adoption of the interventions may increase costs of interventions but Rogowski et al. considered that expected savings and benefits would offset these [24]. The potential for higher cost savings and effectiveness through a wider use or broader scale of QICs is a pertinent aspect of these studies for healthcare budgets.

The establishment of collaboratives was shown to require considerable investment in the initial phases of the improvements, which then decreased over time of the collaborative process. QICs were funded in most studies by national agencies with specialist healthcare improvement staff involved in developing the collaborative, engaging participants and providing education, guidance and support for the duration. Only one study identified the relative cost-effectiveness of different combinations of elements of a QIC [31]. This suggests an opportunity to improve cost-effectiveness of QICs by selecting key elements for uses.

Despite increasing acknowledgement of the importance of patient and public involvement, there was no involvement of members of the public or patients reported in these studies. Costs were spread across state and national healthcare systems to scale up improvements for low per clinic or patient cost. One study included the external cost of developing guidelines in the assessment of cost-effectiveness [35] which provided an additional insight into the costs of developing or adapting international guidelines to national conditions. In most cases the clinical guidelines were developed separately to implementation in healthcare services and funded separately. Despite this inclusion of the cost of developing guidelines, the use of the QIC was shown to be cost-effective [35].

The identified costs of the QIC had similar elements across the five studies showing cost-effectiveness [30, 31, 33, 35, 36]. Costs were highest for the initial development of collaboratives, face to face meetings and travel for participants, and for multi-factored interventions. While most studies used similar components of QICs as described by Nadeem et al. [13] and IHI [14], only one study compared the costs of different elements of the QIC [31]. There is an opportunity to consider which elements of QICs contribute to cost effectiveness and in which setting they may be useful. One study included the cost of development of guidelines and a maintenance cost for an ongoing collaborative [35]. This provides a wider consideration of all set up costs for quality improvement and the costs to maintain the collaborative beyond a research study. The local infrastructure costs varied widely in four studies [31, 34, 36, 37] which made the cost assessments difficult to compare within and between studies. Inclusions and exclusions of costs varied between studies which also made comparisons between studies difficult. It would be of use to identify common costs to consider when budgeting for QICs and to allow for local differences in infrastructure.

The value of these studies shows that savings can be made to healthcare for quality improvements, the real set up costs and how to assess benefit. Caution in interpreting results is needed as the studies varied in what was included and costed and the perspective from which assessment of cost effectiveness was judged. Similarly, few studies of cost effectiveness of QICs were identified suggesting that studies with negative results may not have been published.

A strength of this review is the rigorous and systematic method used to identify studies and synthesise data. A comprehensive search strategy was developed and used in a range of databases. Our search of the grey literature was an important step given the variety of ways in which healthcare improvements are reported. The use of both the CHEERS checklist [24] and Evers CHEC-List [25] to assess the mixed designs found most studies to be of good to medium quality. The main limitations of the review are that only studies published in English were considered and we did not search trial registers. The few papers identified may reflect a publication bias or may indicate economic evaluations of QICs have not been conducted.

## Conclusion

Few cost analyses or cost-effectiveness studies have been identified to assess the costs and benefits of QICs to translate research and knowledge into practice. Most that are included in this review show cost savings or improvement in healthcare process and patient outcomes across acute, long term care and chronic conditions. Judgement is required in relation to the priority given to healthcare improvement from a societal perspective compared to the cost of QICs. The potential to scale up knowledge translation through QICs and to improve cost-effectiveness based on these studies is suggested. The costs of QICs need to be factored into translation of improvements, and their costs or cost-effectiveness evaluated to identify savings to healthcare budgets and benefits to society. A detailed break-down of costs of QICs may assist in identifying elements of greatest cost and alternatives that may be effective for cost savings to the quality improvement process.

## Supplementary information

**Additional file 1.** Medline Search Strategy using medical subject search headings (MeSH) and text words to search for studies and adapted to search other data bases.

**Additional file 2.** Table 1 Overview of studies data extraction: a modified version of JBI data extraction form describing nine aspects of each of the eight studies included in the review.

**Additional file 3.** Table 2a CHEERS Checklist of included economic evaluation studies: A completed checklist of 24 items used to assess the methodological quality of all included studies in the review.

**Additional file 4.** Table 2b Evers Chec-List of quality of full economic evaluations only: A completed checklist of 19 items to assess the methodological quality of full economic evaluations included in the review.

**Additional file 5.** Table 3 JBI Dominance Ranking Matrix: a three by three dominance ranking matrix (DRM) tool to classify the cost-effectiveness results of the included studies as dominant and favoured, unclear or rejected.

**Additional file 6.** Table 4 Costs of aspects of Quality Improvement Collaboratives in the selected studies: a comparison of costs of QICs between eight selected studies by 4 main aspects of cost of QIC

## Data Availability

The datasets used and/or analysed during the current study are available from the corresponding author on reasonable request.

## Abbreviations

€: European Euros
ACE Inhibitor: Angiotensin-converting enzyme inhibitor
CDPC: Cognitive Decline Partnership Centre
CEA: Cost Effectiveness Analysis
CER: Cost Effectiveness Ratio
CHEERS: Consolidated Health Economic Evaluation Reporting Standards statement
CUA: Cost Utility Analysis
DALY: Disability Adjusted Life Year
DRM: Dominance Ranking Matrix
EQ-5D 3 L: European Quality Group 5 dimensions 3 levels measure of quality of life
EVERS CHEC-List: Consensus on Health Economic Criteria checklist by Silvia Evers et al.
ICER: Incremental Cost Effectiveness Ratio
IHI: Institute for Healthcare Improvement
JBI: Johanna Briggs Institute
MeSH: Medical subject search headings
NHMRC: National Health and Medical Research Council, Australia
PRISMA: Preferred Reporting Items for a Systematic Review and Meta-Analysis of Diagnostic Test Accuracy studies
QALY: Quality Adjusted Life Year
QI: Quality Improvement
QIC/s: Quality Improvement Collaborative/s
QoL: Quality of Life
UK: United Kingdom of Great Britain; US/USA: United States/United States of America
