# Supplementary material for: Costs and economic evaluations of Quality Improvement Collaboratives in healthcare: a systematic review
Source: BMC Health Serv Res. 2020 Mar 2;20:155. doi: 10.1186/s12913-020-4981-5 (PMC7053095; doi:10.1186/s12913-020-4981-5)
Supplement: Supplementary file 1 — Additional file 1. Medline Search Strategy using medical subject search headings (MeSH) and text words to search for studies and adapted to search other data bases. [file 12913_2020_4981_MOESM1_ESM.docx]

**Additional file 1**

|  | 1. **MEDLINE search strategy using MeSH headings and text words to search for studies and adapted to search other data bases** |  |
| --- | --- | --- |
| 1. **#** | 1. **Searches** | 1. **Results** |
| 1. 1 | 1. quality improvement/ or value-based insurance/ | 1. 21226 |
| 1. 2 | 1. (quality improvement or QI).ti,ab,kf. | 1. 38432 |
| 1. 3 | 1. quality collaborat*.ti,ab,kf. | 1. 301 |
| 1. 4 | 1. or/1-3 | 1. 51672 |
| 1. 5 | 1. (guidelines as topic/ or practice guidelines as topic/ or Total Quality Management/ or evidence based medicine/ or evidence based practice/) and ("Diffusion of Innovation"/ or Translational Medical Research/ or Cooperative behavior/) | 1. 6246 |
| 1. 6 | 1. ((implementation or diffusion or adher* or follow* or align* or based) adj2 guideline*).tw,kf. | 1. 29179 |
| 1. 7 | 1. or/5-6 | 1. 35186 |
| 1. 8 | 1. or/4,7 | 1. 85363 |
| 1. 9 | 1. Cost-Benefit Analysis/ | 1. 77500 |
| 1. 10 | 1. economics/ or "costs and cost analysis"/ or "cost allocation"/ or "cost control"/ or "cost of illness"/ or health care costs/ or direct service costs/ or hospital costs/ or health expenditures/ or economics, hospital/ or hospital charges/ or economics, medical/ or fees, medical/ or economics, nursing/ or economics, pharmaceutical/ or quality-adjusted life years/ | 1. 206691 |
| 1. 11 | 1. ((cost* or economic*) adj3 (minimi* or utilit* or evaluat* or review* or outcome* or analys* or effect* or benefit)).tw,kf. | 1. 193532 |
| 1. 12 | 1. (CBA or BCA).tw,kf. | 1. 12738 |
| 1. 13 | 1. (marginal analy* or economic impact* or QALY*).tw,kf. | 1. 19571 |
| 1. 14 | 1. or/9-13 | 1. 400949 |
| 1. 15 | 1. 8 and 14 | 1. 5149 |
| 1. 16 | 1. limit 15 to English language | 1. 4935 |
