# Supplementary material for: Costs and economic evaluations of Quality Improvement Collaboratives in healthcare: a systematic review
Source: BMC Health Serv Res. 2020 Mar 2;20:155. doi: 10.1186/s12913-020-4981-5 (PMC7053095; doi:10.1186/s12913-020-4981-5)
Supplement: Supplementary file 2 — Additional file 2. Table 1 Overview of studies data extraction: a modified version of JBI data extraction form describing nine aspects of each of the eight studies included in the review. [file 12913_2020_4981_MOESM2_ESM.docx]

**ADDITIONAL FILE 2**

| **Table 1 Overview of data extraction of included studies** | | | | | |
| --- | --- | --- | --- | --- | --- |
| **Economic Evaluations** | | |  | | |
|  | Broughton et al. 2013,  (30) Niger | Gustafson et al. 2013, (31)  United States of America | Schouten et al. 2010, (33)  The Netherlands | Makai et al 2010, (32)  The Netherlands | Huang et al. 2007, (34)  United States of America |
| Study design and cost perspective | - Cost Effectiveness Analysis (CEA) and Cost Utility Analysis (CUA) of Obstetric and new-born care - Limited to the perspective of National Ministry of Health | - Cost Effectiveness Analysis (CEA) of elements of QIC in addition treatment - Limited to the perspective of National healthcare system | - Cost Utility Analysis (CUA) of a QIC focusing on patients with diabetes - Limited to the perspective of the National Healthcare system | - Cost Utility Analysis (CUA) of QIC to reduce pressure ulcer wounds - Limited to the perspective of the long-term care system | - Cost Utility Analysis (CUA) of QIC to improve diabetes care - Societal perspective of long-term costs |
| Target population | Medical and nursing staff in clinics for the care of women and newborns | Clinical allied health staff and managers of clinics treating people with addictions | Multi-disciplinary teams treating people with type 2 diabetes in outpatient hospital and community clinics | Medical and nursing staff of long- term care of older people in nursing homes to end of life | Medical nursing and administrative staff in Community health centres treating people with chronic health conditions |
| Intervention Comparator | Pre intervention compared to post intervention data for participating clinics | Cluster randomised trial: 4 intervention groups pre, during and post data collection compared to each other | Controlled before and after study in 8 clinics compared to usual care in matching control clinics | Pre intervention cases of pressure ulcers compared to post intervention and to control group in participating care homes | Serial cross section data of 80 randomly selected patients treated reviewed over 4 years |
| Time Horizon / reported cost date/ discount rate | 2 years using US$ 2008 (US $ 2018) and 3% discount rate | 1.5 years using US$ (year and discount rate not stated) | 2 years using Euros 2006 (US $ 2018) and 3% discount rate | 2 years using Euros 2006 (US $ 2018) and 4% discount rate for costs and 1.5% for effects | 4 years using US $ 2004 (US$ 2018) and 3% discount rate for costs and outcomes |
| Model | Monte Carlo simulation and synthesis /decision tree analysis to compare outcomes and costs pre and post intervention. | Drug abuse cost analysis program to measure process improvements. | Dutch Diabetes model (based on Markov model) to calculate life-time medical costs and health outcomes. | Markov decision analytical model | Markov Monte Carlo simulation and synthesis to compare costs, outcomes over 4 time periods |
| Costs included | - Estimates of clinical costs before and after intervention from Ministry of Health user fees and survey of managers - Capital costs per clinic - QI costs of development, capital costs of equipment - Salaries of QI staff consultants, fees, travel, vehicles, fuel. | Cost of personnel, capital, travel, accommodation, data management. | - Diabetes control measures score (UKPDS) - QALY based on the EQ-5D 3L - Numbers of diabetes related health visits, volume and type of medication. | - Program and organisational costs of time spent in training planning and implementation, materials and miscellaneous costs - Cost of materials and equipment used in patient care - Time spent in preventative patient care | - Estimates based on observed services and national use of medications studies for diabetes care, costs of medicines from wholesale drug prices, costs of projected complications - Estimates of cost of the collaboration were based on case studies at Year 1 $712 (US$ 927) pp, Year 2 $600 (US$ 781) pp, Year 3 $ 472 (US$614) pp and Year 4 $378 (US$492) pp - Year 4 costs required for remainder of persons life to sustain benefits |
| Measures of health benefits and cost effectiveness | - Costs per normal delivery, costs of moderate to severe Post-Partum Haemorrhage. | Improvements in retention, waiting times, new patients. Comparison between four interventions. | - Improved blood pressure, high density lipids and cholesterol control - QOL, life expectancy improved and QALYs | - Pre and post differences in preventive measures used - Numbers of pressure ulcer free patients, numbers of Pressure ulcer grades1-4 and mortality, QoL - Sustainability of prevention | - Improved processes of screening and prescribing - Reduced intermediate and end stage complications - Improved QALYs |
| Conclusions | - Decreased average cost per delivery from $35 ($41) to $28 ($32.8) - Incremental cost of QI per delivery $2.43 ($ 2.84) - QI modest cost per delivery and decreased average cost per delivery - 89% decrease in PPH - Overall cost saving and improved health outcomes | - Coaching and combination of collaborative elements produces significant improvements in waiting time and new patients. No significant effect on retention - Coaching is more cost effective to achieve improvement | - QIC is cost effective - Implementation costs €**22** per patient. (US $19.5) | - Reduced prevalence of pressure ulcers - Increase in healthcare costs overall - Uncertainty in cost effectiveness of the QIC due to the end of life stage of patient population - Cost effectiveness likely if results sustained | - Diabetes HDC program is cost effective compared to other healthcare technology - Effectiveness of individual elements of care varied widely but greatest health benefits in lowering glucose levels and increasing ACE inhibitors - Costs borne by health centres and health insurance |
| ICER / overall result | - Estimated ICER $286 ($335) per DALY - 2.6% decrease in DALY’s for clinics post collaborative improvement. - 3% decrease ICER between pre and post incidence of PPH. | - CER of US$0.56 (no base date given) per patient per waiting day saved in the coaching group compared to CER of US$37.30 per patient per day saved for the combination group. No statistically significant effect found for retention of patients | - ICER €1937 (US $1,714.5 for men and €1751 (US $1,550 for women per QALY compared to usual care. - Probability of collaborative being cost effective using threshold of €20, 000 (US $17,703) per quality adjusted life year was >95% | - ICER €78,517 (US$ 69,598) per QALY for most sustained change - ICER €88,692 (US $78,504) per QALY for partially sustained change and - ICER €131,253 (US$116,176) per QALY for not sustained with threshold of ICER €80,000 (US $70,810) per QALY for patients with high disease severity | - ICER use of ACE inhibitor $26,653 (US$ 23,702) per QALY - ICER for individual therapies were not cost effective - Multiple processes of care improved and led to lower lifetime incidence of complications - Overall ICER $54,060 (US$ 70,386) per QALY with program costs of $100 (US$130) per patient per year |
| **Cost Analyses** | | | | | |
|  | Bloem et al. 2017, (35)  The Netherlands | Dranove et al. 1999, (37)  United States of America | Rogowski et al. 2001, (36)  United States of America |  |  |
| Study design and cost perspective | - Cost Analysis - Limited to the perspective of the National Healthcare system | - Cost Analysis - Limited to the perspective of the National Health care system | - Cost Analysis - Limited to the perspective of the National Health care system |  |  |
| Target population | Allied health clinicians treating people diagnosed with Parkinson’s Disease in community | Hospital staff involved in QI related to hip replacement and coronary care of adult patients | Staff of neonatal intensive care clinics treating infants with low birth weight and lung infections |  |  |
| Intervention Comparator | 9 non-randomised control services not in ParkinsonNet | Comparison of cost of QI with patient outcomes and condition specific costs to hospitals without QI | Comparison of costs between intervention hospitals and control hospitals for treatment and pre and post costs in intervention hospitals |  |  |
| Time horizon/ reported cost date | 5 years / US$ 2017 (US$ 2018) | Not reported / US$ 1997 (UD$ 2018) | 2 years US$ 1996 (US$ 2018) |  |  |
| Model | - Comparison of cost savings per person to cost of network per capita over 5 years - Comparison of costs of network staff to usual care | Correlation between direct costs and meeting costs of QI compared to patient costs and outcomes | Pre-post statistical comparison of treatment costs to test for significance between control and intervention groups |  |  |
| Costs included | - Medical claims data for patients with PD and caregivers, total annual medical costs pa per patient with PD - Set up costs of network and maintenance costs over 5 yrs. | Costs of QI per hospital admission | - Hospital Treatment cost per infant from hospital bills - Costs of staff time spent in meetings education, reporting collecting data, travel costs conference calls, benchmark costs, data costs, survey |  |  |
| Measures of health outcomes/ cost effectiveness | - Reduced treatment sessions, reduced disease complications, reduced dependence on medical care - Infrastructure and personnel costs, annual maintenance costs of network | QI costs per hospital: meeting costs, training, accreditation, personnel costs, overheads, consultants’ fees | Treatment cost per infant and resources spent by hospital on QIC. |  |  |
| Conclusions | - Modest cost savings per patient with PD of US $439 (US$ 449) pa or 5% of expenditure per patient with PD pa - Cost of Parkinson Net spread over 5 years was US$29   (US$ 30) per patient pa   - Savings expected for total population in Netherlands with PD far outweigh costs of the ParkinsonNet | Wide variation in costs of QI across hospitals and no significant correlation between mature CQI hospitals and immature hospital CQI programs | - Average cost savings in treatment far outweighed the cost of the QIC. Savings of $14 (US$ 21) million for a cost of $1.5 (US$ 2.3) million investment - Wide variations in costs and savings. |  |  |
| Overall result | Potential cost savings pa in Netherlands between US$17-$66 (US$ 17.4-67.5) million pa | No correlation between costs per hospital for CQI and outcome | Average cost savings far outweigh the cost of the QIC and was sustainable in the short term |  |  |
| Abbreviations used: QI: quality improvement, QIC: quality improvement collaborative, MoH: Ministry of Health in Niger, HDL: high density lipids, PPH: post-partum haemorrhage. ICER: incremental cost effectiveness ratio, DALY: disability-adjusted life year, NIAT: network for the improvement of addiction treatment, QALY: quality adjusted life years, UKPDS: United Kingdom Prospective Diabetes Score, CQI: continuous quality improvement, PD: Parkinson’s Disease, pa: per annum, US$: United States Dollars, €: European Euros. All currencies converted to US$ 2018. | | | | | |
