# Supplementary material for: Costs and economic evaluations of Quality Improvement Collaboratives in healthcare: a systematic review
Source: BMC Health Serv Res. 2020 Mar 2;20:155. doi: 10.1186/s12913-020-4981-5 (PMC7053095; doi:10.1186/s12913-020-4981-5)
Supplement: Supplementary file 3 — Additional file 3. Table 2a CHEERS Checklist of included economic evaluation studies: A completed checklist of 24 items used to assess the methodological quality of all included studies in the review. [file 12913_2020_4981_MOESM3_ESM.docx]

| **ADDITIONAL FILE 3**  **Table 2a. CHEERS checklist of quality of included economic evaluation studies** | | | | | | | | |
| --- | --- | --- | --- | --- | --- | --- | --- | --- |
| Items | Broughton  et al. 2013, (30) | Gustafson  et al. 2013, (31) | Schouten et al. 2010, (33) | Bloem et al. 2017, (35) | Dranove et al. 1999, (37) | Rogowski et al. 2001, (36) | Makai et al.  2010, (32) | Huang et al. 2007, (34) |
| 1.Identified as  Economic evaluation | **Y CEA and CUA** | **N within-study CEA** | **Y model-based CUA** | **N** | **N** | **N** | **Y**  **CUA** | **Y model-based CUA** |
| 2.Structured Summary/ Abstract | **Y** | **Y** | **Y** | **N** | **Y** | **Y** | **Y** | **Y** |
| 3.Broader context for study stated, question and relevance | **Y** | **Y** | **Y** | **Y** | **Y** | **Y** | **Y** | **Y** |
| 4.Characteristics of population and subgroups, why chosen | **Y** | **Y** | **Y** | **Y** | **Y** | **Y** | **Y** | **Y** |
| 5.Setting and location stated | **Y** | **Y** | **Y** | **Y** | **Y** | **Y** | **Y** | **Y** |
| 6.Study perspective related to costs | **Y** | **Y** | **Y** | **Y** | **Not stated** | **Not stated** | **Y** | **Y** |
| 7.Comparators stated and why | **Y** | **Y** | **Y** | **Y** | **Y** | **Y** | **Y** | **Y** |
| 8.Time horizon and why chosen | **Y** | **Y** | **Y** | **Y** | **N** | **Y** | **Y** | **Y** |
| 9.Discount rate used and why chosen | **Y** | **N** | **Y** | **N** | **N** | **N** | **Y** | **Y** |
| 10.Choice of health outcomes and relevance to analysis | **Y** | **Y** | **Y** | **Y** | **N** | **Y** | **Y** | **Y** |
| 11. Measure of effectiveness  b) SYNTHESIS based study | **Y** | **Y** | **Y** | **N** | **N** | **N** | **Y** | **Y** |
| 12.Measure and Valuation of preferences for outcomes | **N** | **N/A** | **N/A** | **N** | **N** | **N** | **N** | **N** |
| 13.Estimating resources and costs described b) model based economic evaluation | **Y** | **N** | **Y** | **N** | **N** | **N** | **Y** | **Y** |
| 14. Currency date and conversion | **Y** | **Y** | **Y** | **Y** | **N** | **Y** | **Y** | **Y** |
| 15. Choice of model for analysis | **Y** | **N** | **Y** | **N** | **N** | **N** | **Y** | **Y** |
| 16. Assumptions described | **Y** | **N** | **Y** | **N** | **Y** | **N** | **Y** | **Y** |
| 17. Analytical methods described | **Y** | **N** | **Y** | **Y** | **Y** | **Y** | **Y** | **Y** |
| 18. Study parameters reported | **Y** | **N** | **Y** | **Y** | **N** | **N** | **Y** | **Y** |
| 19. Incremental costs and outcomes, means and difference | **Y** | **Y** | **Y** | **N** | **N** | **N** | **Y** | **Y** |
| 20 Uncertainty described b) model based | **N** | **N** | **Y** | **N** | **N** | **N** | **Y** | **Y** |
| 21. heterogeneity differences in cost or variability | **Y** | **Y** | **Y** | **N** | **N** | **N** | **Y** | **Y** |
| 22. study findings | **Y** | **Y** | **Y** | **Y** | **Y** | **Y** | **Y** | **Y** |
| 23. source of funding | **Y** | **Y** | **Y** | **Y** | **Y** | **Y** | **Y** | **Y** |
| 24. conflicts of interest | **N** | **Y** | **Y** | **Y** | **N** | **N** | **Y** | **Y** |
| Summary score | **22/24** | **15/24** | **23/24** | **13/24** | **9/24** | **11/24** | **23/24** | **23/24** |
