# Supplementary material for: Costs and economic evaluations of Quality Improvement Collaboratives in healthcare: a systematic review
Source: BMC Health Serv Res. 2020 Mar 2;20:155. doi: 10.1186/s12913-020-4981-5 (PMC7053095; doi:10.1186/s12913-020-4981-5)
Supplement: Supplementary file 4 — Additional file 4. Table 2b Evers Chec-List of quality of full economic evaluations only: A completed checklist of 19 items to assess the methodological quality of full economic evaluations included in the review. [file 12913_2020_4981_MOESM4_ESM.docx]

**Additional File 4**

| **Table 2b. Evers CHEC-List (21) of quality of full economic evaluations only** | | | | | |
| --- | --- | --- | --- | --- | --- |
|  | Broughton et al. 2013, (30) | Gustafson et al. 2013, (31) | Schouten et al. 2010, (33) | Makai et al. 2010, (32) | Huang et al. 2007, (34) |
| 1. Is the study population clearly described? | **y** | **y** | **y** | **Y** | **Y** |
| 2. Are competing alternatives clearly described? | **y** | **y** | **y** | **Y** | **Y** |
| 3. Is a well-defined research question posed in answerable form? | **y** | **y** | **y** | **Y** | **Y** |
| 4. Is the economic study design appropriate to the stated objective? | **y** | **y** | **y** | **Y** | **Y** |
| 5. Is the chosen time horizon appropriate to include relevant costs and  consequences? | **y** | **y** | **y** | **Y** | **Y** |
| 6. Is the actual perspective chosen appropriate? | **y** | **y** | **y** | **Y** | **Y** |
| 7. Are all relevant costs for each alternative identified? | **y** | **y** | **y** | **Y** | **Y** |
| 8. Are all costs measured appropriately in physical units? | **y** | **y** | **y** | **Y** | **Y** |
| 9. Are all costs valued appropriately? | **y** | **y** | **y** | **Y** | **Y** |
| 10. Are all important and relevant outcomes of alternatives performed? | **y** | **y** | **y** | **Y** | **Y** |
| 11. Are all outcomes measured appropriately? | **y** | **y** | **y** | **Y** | **Y** |
| 12. Are outcomes valued appropriately? | **y** | **y** | **y** | **Y** | **Y** |
| 13. Is an incremental analysis of costs and outcomes of alternative  performed? | **y** | **y** | **y** | **Y** | **Y** |
| 14. Are all future costs and outcomes discounted appropriately? | **y** | **n** | **y** | **Y** | **Y** |
| 15. Are all important variables, whose values are uncertain, appropriately  subjected to sensitivity analysis? | **y** | **n** | **y** | **Y** | **Y** |
| 16. Do the conclusions follow from the data reported? | **y** | **y** | **y** | **Y** | **Y** |
| 17. Does the study discuss the generalisability of the results to other  settings and patient and client groups? | **y** | **y** | **y** | **Y** | **Y** |
| 18. Does the article indicate that there is no potential conflict of interest of  study researcher and funder? | **N** | **N** | **y** | **Y** | **Y** |
| 19. Are ethical and distributional issues discussed appropriately? | y | N | Y | Y | Y |
| Summary score | 18/19 | 15/19 | 19/19 | 19/19 | 19/19 |
