# Supplementary material for: Costs and economic evaluations of Quality Improvement Collaboratives in healthcare: a systematic review
Source: BMC Health Serv Res. 2020 Mar 2;20:155. doi: 10.1186/s12913-020-4981-5 (PMC7053095; doi:10.1186/s12913-020-4981-5)
Supplement: Supplementary file 5 — Additional file 5. Table 3 JBI Dominance Ranking Matrix: a three by three dominance ranking matrix (DRM) tool to classify the cost-effectiveness results of the included studies as dominant and favoured, unclear or rejected. [file 12913_2020_4981_MOESM5_ESM.docx]

**ADDITIONAL FILE 5**

| **Table 3 JBI Dominance Ranking Matrix (33)** | | | |
| --- | --- | --- | --- |
| Cost | Health benefit | Implication for decision-makers | No. of studies |
| + | - | Reject intervention |  |
| 0 | - | Reject intervention |  |
| + | 0 | Reject intervention | 1 (Dranove et al. (37)) |
| - | - | Unclear – Judgment required on whether intervention preferable considering incremental cost-effectiveness measures and priorities/willingness to pay |  |
| 0 | 0 | Unclear – Judgment required on whether intervention preferable considering incremental cost -effectiveness measures and priorities/willingness to pay |  |
| + | + | Unclear – Judgment required on whether intervention preferable considering incremental cost-effectiveness measures and priorities/willingness to pay | **2 (Huang et al. (34) and**  **Makai et al. (32))** |
| - | 0 | Favour intervention | **2 (Gustafson et al. (31) and**  **Rogowski et al (36)**) |
| 0 | + | Favour intervention |  |
| - | + | Favour intervention | **3 (Bloem et al.(35),**  **Broughton et al. (30) and**  **Schouten et al. (33))** |
