# Supplementary material for: Costs and economic evaluations of Quality Improvement Collaboratives in healthcare: a systematic review
Source: BMC Health Serv Res. 2020 Mar 2;20:155. doi: 10.1186/s12913-020-4981-5 (PMC7053095; doi:10.1186/s12913-020-4981-5)
Supplement: Supplementary file 6 — Additional file 6. Table 4 Costs of aspects of Quality Improvement Collaboratives in the selected studies: a comparison of costs of QICs between eight selected studies by 4 main aspects of cost of QIC [file 12913_2020_4981_MOESM6_ESM.docx]

**ADDITIONAL FILE 6**

| **Table 4. Costs of aspects of Quality Improvement Collaboratives in selected studies** | | | | | | | | |
| --- | --- | --- | --- | --- | --- | --- | --- | --- |
|  | Bloem et al 2017, (35) | Broughton et al 2013, (30) | Dranove et al 1999, (37) | Gustafson et al 2013, (31) | Rogowski et al 2001, (36) | Schouten et al 2010, (33) | Makai et al 2010, (32) | Huang et al 2007, (34) |
| Set up and maintenance of QIC, personnel, experts, training, regional support, promotion | - 2017 US$ 2.24 Million (US$2.3M 2018) national network over 5 years - 2017 USD1.5 million (US$1.54M 2018) maintenance of network | - 2008 US$ 188,400 (US$221,000 2018) development costs - 33 hospitals over 3 years | 1998 US$ 1.1 million (US$1.6 M 2018) mean costs over 16 hospitals per annum | 2011 US$1.6 million (US$1.8 M 2018) for 201 clinics across 5 states over 2 years | 1996 US$ 820,000 (US$1.14M 2018) 10 hospitals over 3 years | 2006 € 261,500 (US$293,902 2018) national over 6 regions and 50 clinics over 1 year | 2006 €50,000 (US$56,4902018) project materials and $64,000 (US$72,306 2018) for collaborative costs | Not provided  Estimates of between 2004 US $712/yr1- $378 yr4 (US$928/yr1- $493/yr4 2018) per patient for the QIC |
| Staff time and travel to participate in meetings, education, capital costs | Not provided | 2008 US$ 583,000 (US$684,000 2018) | 1998 US$250,000 (US$367,0002018) mean included in the per hospital mean costs | 2011 US$ 804,915 (US$907,000 2018)  For all groups | 1996 US$682,060 (US$945,000 2018) | 2006 €381,604 (US$431,129 2018) | Travel costs not separated | Not provided |
| IT Costs | 2017 US$ 346,000 (US$354,427 2018) | Not identified | Not identified | Not identified | Not identified | Not identified | Not identified | Not provided |
| Evidence-based guideline development | 2017 US$ 777,000 (US$796,000 2018) for 9 guidelines approx. $75,000ea. (US$76,800 2018) | Not identified | Not identified | Not identified | Not identified | Not identified | Not identified | Not identified |

All currencies converted to US$ 2018
